# *Rosa* × *damascena* Herrm. From Azaran region, Kashan: rich in saturated and unsaturated fatty acids with inhibitory effect against *Proteus mirabilis*

**DOI:** 10.1186/s12906-024-04562-7

**Published:** 2024-07-09

**Authors:** Mansureh Ghavam

**Affiliations:** Department of Nature Engineering, Faculty of Natural Resources and Earth Sciences, University of Kashan, Kashan, Iran

**Keywords:** Essential oil, *Rosa*, Fatty acids, Inhibitory activity, Kashan

## Abstract

**Background:**

One of the most widely used medicinal plants in Iranian traditional medicine, *Rosa* × *damascena* Herrm. (mohammadi flower) that the people of Kashan use as a sedative and to treat nervous diseases and constipation. In this research, the yield, chemical composition and antimicrobial activity of the essential oil of this plant were evaluated for the first time from Azaran region, Kashan.

**Methods:**

The essential oil was extracted by means of hydrodistillation (Clevenger), and its chemical compounds were identified and determined by GC/MS. The antimicrobial activity of the essential oil was determined by the diffusion method in agar, the minimum growth inhibitory concentration (MIC) and the minimum concentration capable of killing bacterial/fungal microorganisms (MBC/MFC).

**Results:**

The results showed that the yield of essential oil was 0.1586 ± 0.0331% (w/w). Based on the results of the chemical composition analysis of *R. x damascena* essential oil, 19 different compounds (98.96%) were identified. The dominant and main components of the essential oil were oleic acid (48.08%), palmitic acid (15.44%), stearic acid (10.17%), citronellol (7.37%) and nonadecane (3.70%). Based on the results of diffusion in agar, the highest zone of inhibition against *Candida albicans* (ATCC 10231) was ~ 9.5 mm. The strongest inhibitory activity of *R. x damascena* essential oil against Gram-negative *Proteus mirabilis* (ATCC 43071) was with the diameter of the inhibition zone (~ 9 mm), which was equal to the strength of rifampin (~ 9 mm).

**Conclusion:**

Therefore, this essential oil is a promising natural option rich in fatty acids, which can be a potential for the production of natural antimicrobials against infectious diseases, especially urinary tract infections.

## Introduction

Rosaceae is one of the diverse and abundant families in terms of plant species. The *Rose* genus is the most valuable genus in the Rosaceae family [1]. The genus *Rosa* includes more than 200 shrub and perennial species, whose medicinal species have fragrant flowers [2]. *Rosa* species are of global economic importance. They are mainly known for their ornamental, medicinal, cosmetic and culinary properties. Rosa flowers are a source of natural aromas and flavors and are used to produce healthy functional foods such as tea, rose water, jam, syrup, jelly, and candy [3].

Damascus rose *Rosa* × *damascena* Herrm. (a hybrid between *R. gallica* L. and *R. moschata* J. Herm. or *R. phoenicia* Boiss) is one of the most productive commercial species due to the superior quality of its essential oils [4, 5]. It has been reported to ethnobotanically treat inflammatory conditions and mental disorders [6]. Due to its many phenolic compounds, the essential oil of this plant has antibacterial and antioxidant properties and is used in various oral, inhaled and topical forms in aromatherapy, treatment of skin diseases, chromosomal mutations and cancer [7]. Damascus rose petal essential oil has an economic value and is widely used in the pharmaceutical, food, cosmetics, perfume and cologne industries, and natural and aromatic products in traditional medicine [8].

In Iran, it is known as "Mohammadi flower" and people believe that Mohammadi flower reminds them of Prophet Muhammad (PBUH) [9]. This plant is one of the most used medicinal plants in traditional Iranian medicine. An Iranian manuscript from the tenth century described the febrifuge, obstructive, and cholestatic effects of *R.* × *damascena* [10]. Its flowers, petals and fruits are traditionally used to treat insomnia in Iran [6]. Ethnobotanical studies in Iran indicate that the people of Raz and Jergalan cities, in the northern and northwestern parts of North Khorasan province, use the decoction and powder of flowers for laxatives, flatulence, sedatives, heart and blood vessels in oral form [11]. The people of Meshkin Shahr, Ardabil province call it "Qezil Gul" and use the essential oil and powder of the flowers for sedative, laxative, memory enhancement, skin softness [12]. The people of Kashan city use as a sedative and to treat nervous diseases and constipation [13]. The people of Darab city use for relaxation [14]. The people of Semnan city use the flower tea of this plant to strengthen the heart and relieve stress. [15]. The people of Baft city of Kerman province use flowers as a flavoring, weak laxative and preparation of rose water [16].

The chemical compounds of *R. x damascena* essential oil, which cause its aroma and medicinal properties, mainly include alcohols (2-henyl ethyl alcohol, geraniol, nerol, and citronellol) and long-chain hydrocarbons such as tricosane, henicosan, eicosane, and nonadecane [17, 18]. It has been reported that the lower the level of hydrocarbons, the better the quality of *R. x damascena* essential oil and their role in the stability of the essential oil aroma [19]. Despite the large selection of synthetic drugs in the pharmaceutical market, *R.* × *damascena* essential oil has not lost its popularity as a source of biologically active substances [20].

The chemical composition of *R.* × *damascena* essential oil varies considerably due to diverse climatic conditions and soil compositions of the fields [21].

Studies indicate that about 400 compounds have been identified in the essential oil of *R. x damascena* according to its ecotypes and species, and new compounds with new properties are always identified in it [22]. As far as we know, the essential oil of *R.* × *damascena* from Azran region has not been fully analyzed and tested before. Considering the traditional uses of Kashan people and the potential pharmacological effects of this species, identifying its chemotype is of key importance, therefore, for the first time, the quantitative chemical composition and antimicrobial activity of the essential oil of *R. x damascena* cultivated in Aazran region were evaluated.

## Materials and methods

### Plant sampling

Sampling of *R.* × *damascena* flowers were collected from three random points from different bases (150 bases) from Aazran region, Kashan, Iran (N 33˚ 43.33ʹ 10ʺ and E 51˚ 8.66ʹ 00ʺ and with an altitude of 2416 m above sea level) in June 2022, coinciding with the opening of the buds at 6 o'clock in the morning (Permission for collection of plant materials obtained from the Agricultural Jahad Office). The flowers were taken to the laboratory and kept at 4°C for one hour. Also, a complete plant sample was collected and after identification, it was registered and kept in the herbarium of Faculty of Natural Resources and Earth Sciences, University of Kashan, Kashan. The plant was identified by Mansureh Ghavam and recorded with code number 1312.

### Extraction, separation and determination of essential oil yield

First, 350 g of fresh flowers from each harvest point were poured into 2-L flask and added to the double-distilled water; so that a total of two-thirds of the flask is filled. Then, the flask was connected to the Clevenger device and the essential oil was extracted by water distillation for 4 h. The extracted essential oil was separated by *n*-pentane (Merck, Germany). After evaporating *n*-pentane, the essential oil was extracted with sodium sulfate and stored in dark and closed bottles, away from light, at 4°C until use in the next step.

Equation 1 (Azernivand et al., 2010) was used to determine the yield of essential oil and was calculated based on weight percentage (w/w). Then the yield of essential oil was reported as mean ± standard deviation.

1 $$100\text{x }(\text{weight of dry plant}/\text{weight of essential oil}) =\text{ yield of essential oil}$$

### Identification and separation of essential oil components

A chromatograph (model 6890) coupled with a mass spectrometer (model N-5973 manufactured by Agilent) (GC/MS), University of Kashan, Iran was used to identify and separate the constituents of the essential oil. A capillary column (HP-5MS) with a 5% methylphenylsiloxane static phase (length 30 m, internal diameter 0.25 mm, static layer thickness 0.25 μm) and ionization energy of 70 eV was used. The temperature program included 60–247 °C with a temperature increase rate of 3 °C/min. The injector and the detector temperatures were set at 250 °C. The ionization energy was 70 eV. The sample injection volume was 1μL with the split ratio (1:50). The flow rate of the helium carrier gas was 1.5 mL/min. Retention indices (RI) were calculated for all chemical compounds using homologous series of C8-C28 n-alkanes. Retention indices were calculated for each peak of the GC–MS spectrum to identify the compounds. The calculated retention indices were compared with tabulated Adams indices stored in the NIST mass spectral database. Also, the mass spectrum was examined to identify the compounds and the identifications were confirmed based on the standard compound mass spectrum and various library sources (Wiley-14 and NIST-14). The relative percentage of each constituent of the essential oil was obtained for the area under the curve in gas chromatography [23].

### Antimicrobial activity of essential oil

#### Preparation and cultivation of standard microbial strains

The standard microbial strains used in this study were obtained from Iran Scientific and Industrial Research Organization (IROST). Bacterial strains include Gram-positive *Staphylococcus epidermidis* (CIP 81.55), *Staphylococcus aureus* (ATCC 29737) and *Bacillus subtilis* (ATCC 6633) and seven Gram-negative bacteria *Klebsiella pneumonia* (ATCC 10031), *Escherichia coli* (ATCC 25922), *Pseudomonas aeruginosa* (ATCC 27853), *Salmonella paratyphi-A serotype* (ATCC 5702), *Shigella dysenteriae* (PTCC 1188) and *Acinetobacter baumannii* (ATCC BAA-747), *Proteus mirabilis* (ATCC 43071) were cultured in Nutrient Agar medium at 37°C for 24 h were incubated in the incubator. A yeast strain of *Candida albicans* (ATCC 10231) was incubated in Sabouraud Dextrose Agar medium at 30°C for 24 h in an incubator.

#### Agar diffusion method

For this purpose, 100 µL of bacterial suspensions (with a turbidity equal to 0.5 McFarland) and yeast were cultured on Mueller Hinton Agar (MHA) and Sabro Dextrose Agar (SDA) culture medium under uniform conditions. Using a Pasteur pipette, wells were created in the culture medium with a regular distance from each other and a suitable distance from the plate wall with an approximate diameter of 6 mm. The essential oil was also dissolved in dimethylsulfoxide (DMSO) and reached a concentration of 300 μg/mL, and about 10 μL of essential oil was inoculated into the wells. Plates were incubated for 48 h at 30°C for yeast and 24 h at 37°C for bacteria. The diameter of the essential oil inhibition zone for each microorganism was determined by measuring with an antibiogram ruler (in millimeters) [[24]]. The experiment was repeated three times.

#### Minimum Inhibitory Concentration (MIC) and Minimum Bactericidal/Fungicidal Concentration (MBC/MFC)

At first, the initial concentration of essential oil was 8000 µg/ml. Then concentrations of 4000, 2000, 1000, 500, 250, 125 and 62.5 μg/mL were prepared from the initial concentration. A sterile 96-well microplate was prepared and 95 µl of brain heart infusion (BHI) broth for bacteria and Sabouraud dextrose (SD) broth for yeast were added to each well of the microplate. 5 µL of microbial suspension with 0.5 McFarland dilution and 100 µL of one of different concentrations of essential oil were added. Plates inoculated with bacterial strains at 37°C for 24 h and plates inoculated with yeast at 30°C for 48 h were heated in an incubator. Microbial growth was determined by the presence of turbidity at the bottom of the well and MIC or minimum inhibitory concentration was determined.

5 µL from each of the microplate wells in which there was no growth (bright well) were inoculated into nutrient agar medium and heated at 37°C for 24 h. Any of the concentrations that did not grow after 24 h killed the microbial strain and the lowest concentration was considered as the minimum concentration of bacterial/fungal lethality [24]. The experiments were repeated three times.

#### Positive control

Antibiotics gentamicin (10µg/disc) and rifampin (5µg/disc) for bacteria and nystatin (100,000 unit/ml) for yeast were used for positive control under the same conditions as the essential oil test.

### Statistical analysis

Statistical analysis was done using SPSS 22 software. After ensuring the normality of the data, the statistical significance of the differences was evaluated using the One-Way Analysis of Variance method. Comparison of means was performed using Duncan's test with a 1% error probability level. All data were expressed as mean ± standard deviation.

## Results

### Color and quantity of essential oil (yield)

The *R.* × *damascena* essential oil was white in color. The color of this essential oil is reported to be pale yellow from the plains of Kashan [25]. By changing the environmental conditions of the plant habitat, the type and percentage of essential oil compounds are affected and this causes the color of the essential oil to change.

Based on the results, the yield of *R.* × *damascena* essential oil was 0.1586% ± 0.0331. The highest yield of this essential oil in Kashan, from Kamoo region, was recorded at 0.1340% [26]. Yousefi and Jaimand [27] reported the difference in the yield of this essential oil in different regions of Iran and confirmed that the average yield of *R. x damascena* essential oil was about 0.01% and the highest yield belonged to Isfahan province (0.018%). Yavari et al. [28] recorded the yield of *R.* × *damascena* essential oil from Saadat region of Fars province, Iran at different harvest times from 0.046% to 0.082% (w/w). Considering the characteristics of the place of growth and the location of the plant in nature is one of the main factors that can have a great impact on the amount of essential oils and effective substances of plants. There have been reports that there is a relationship between habitat conditions and the chemical compounds of plants, and a high correlation between the geographical origin of plants and the amount of effective compounds has been shown [29, 30].

### Essential oil quality based on GC/MS results

**Fig. 1:**
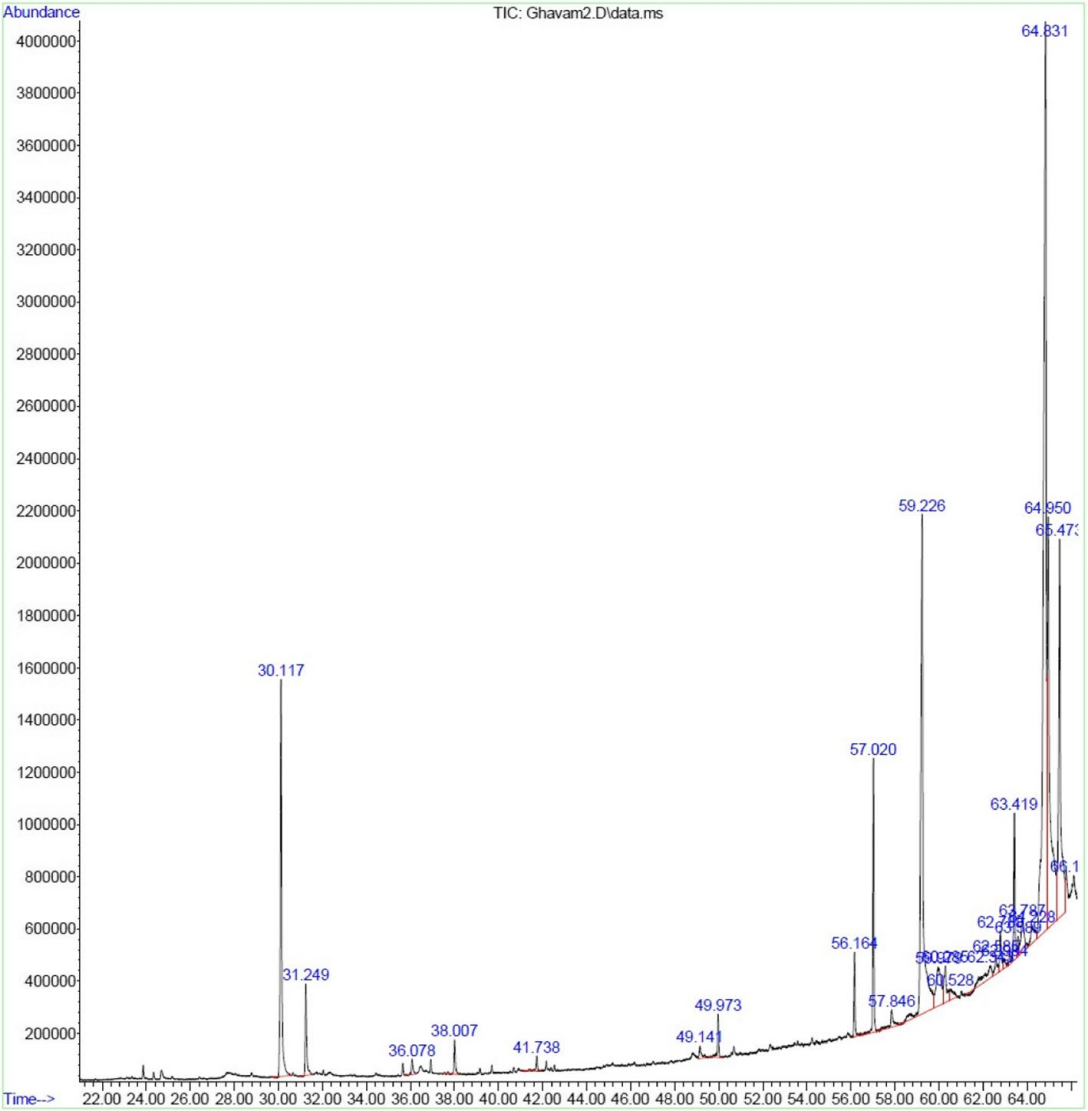
Representative GC–MS chromatogram of *R. x damascena* essential oil

Based on the results of the analysis of the chemical compounds of *R. x damascena* essential oil using GC/MS, 19 different compounds were identified, which included 98.96% (Table 1 and Fig. 1). Similarly, Davoodi, et al. (2019) [31] reported 19 compounds (90.3–99.9%) from different regions of Chaharmahal and Bakhtiari provinces of Iran for this essential oil. Hadipour et al. [32] 63 compounds (99.8%) from Kashan, Yavari et al. [28] 30 compounds (97.6%) from Saadat Shahr of Fars province, Shahbazi et al. [33], 23 compounds (97.81%) from different regions of Iran, and [34], 23 compounds (98.66%) from Darab in Fars province, which is contrary to the present results. It seems that the difference in the habitat conditions of a species has caused the difference in the number of compounds that make up this essential oil in different regions of Iran. Environmental conditions may affect chemical profiles. Furthermore, genetic differences and natural selection events can partially explain chemical diversity [35].

**Table 1:** Chemical compositions of *R. x damascena* essential oil

o	Compound	. RI _Exp_	RI _Lit_	Mean (%)	Molecular formula	Identification
1	Citronellol	1292.534	1245	7.37	C_10_H_20_O	MS, RI, AU
2	Geraniol	1317.656	1267	1.58	C_10_H_18_O	MS, RI, AU
3	Eugenol	1427.343	1359	0.29	C_10_H_12_O_2_	MS, RI
4	Methyleugenol	1473.082	1399	0.57	C_11_H_14_O_2_	MS, RI
5	Germacrene D	1564.858	1519	0.33	C_15_H_24_	MS, RI
6	1-Heptadecene	1761.009	1690	0.34	C_17_H_34_	MS, RI
7	Heptadecane	1786.376		0.69	C_17_H_36_	MS, RI
8	9-Nonadecene	1883.725	1893	1.15	C_19_H_38_	MS, RI
9	Nonadecane	1896.497		3.70	C_19_H_40_	MS, RI
10	cis-13-Octadecenoic acid	1917.891		0.70	C_18_H_34_O_2_	MS, RI
11	Hexadecanoic acid = Palmitic acid	1959.720	1964	15.44	C_16_H_32_O_2_	MS, RI, AU
12	9-Octadecenoic acid (Z)- = Oleic cid	1982.624	2140	48.08	C_18_H_34_O_2_	MS, RI, AU
13	Eicosane	1992.010		1.22	C_20_H_42_	MS, RI
14	9-Hexadecenal	1999.301	1759	0.55	C_16_H_30_O	MS, RI, AU
15	9-Octadecenal, (Z)-	2032.199	2010	1.13	C_10_H_12_O_3_	MS, RI, AU
16	9,12-Octadecadienoic acid (Z,Z)-, methyl ester = Linoleic acid, methyl ester	2089.693	2092	1.99	C_19_H_34_O_2_	MS, RI, AU
17	9,17-Octadecadienal	2093.162	2297	1.08	C_18_H_32_O_2_	MS, RI
18	Heneicosane	2589.578		2.27	C_21_H_44_	MS, RI, AU
19	Octadecanoic acid = Stearic acid	2656.666	2188	10.17	C_18_H_36_O_2_	MS, RI, AU
	Total			100		
	Monoterpenes hydrocarbons			6.7		
	Oxygenated monoterpenes			47.87		
	Sesquiterpenes hydrocarbons			2.07		
	Oxygenated sesquiterpenes			5.52		
	Others (Nonterpenoids)			38.66		

Compounds are listed in order of their retention time from an HP-5 column. RI _Exp_., linear retention indices on HP-5 column, experimentally determined using homolog series of n-alkanes (C8-C28). RI _Lit._, Linear retention index taken from [70], or NIST 14 (2014) and literature. Identification method: compounds were identified by comparison of their mass-spectral data (MS) and retention indices (RI) with those of the Wiley Registry of Mass Spectral Data (2014), NIST Mass Spectral Library (2014), and the literature; for some compounds, the identification was confirmed by coinjection with authentic compound (AU)

In this essential oil, nonterpenoids group constituted the highest percentage of compounds with 89.37%, and monoterpenes hydrocarbons and oxygenated sesquiterpenes were not observed at all. Similarly, the absence of oxygenated sesquiterpenes and the predominance of nonterpenoids have been reported from Isfahan province with an amount of 80.66% and from Sefidshahr of Kashan with an amount of 68.15% for this essential oil [25, 36]. The highest amount of nonterpenoids group was reported from Sedeh region of Kashan with the amount of 70.02% [26]. Previous studies indicate that in some regions of Iran, oxygenated monoterpenes were mainly the dominant group of this essential oil, and nonterpenoids were in second place [28]

The ANOVA results showed that there was a significant difference between the average amount of different components obtained from *R. damascena* essential oil (*P* ≤ 0.01). 9-Octadecenoic acid (Z)- or oleic acid with the amount of 48.08% was the dominant and main composition of this essential oil. Based on previous studies and to the best of our knowledge, there is only one report of the presence of this essential oil in Iran, which is related to the sample collected from Isfahan (17.63%) [36]. Oleic acid is the most abundant monounsaturated fatty acid in the human diet and in the human circulatory system [37]. In the brain, it is a major component of membrane phospholipids and is highly abundant in myelin [38]. A significant decrease in oleic acid has been observed in the brains of patients with major depressive disorder and Alzheimer's disease [39]. It has many biological effects such as antibacterial effects [40], hypocholesterolemia [41], antioxidant [42], prevention of atherosclerosis, anti-inflammatory [43], and protective effect on breast cancer and improves the function of the immune system [44].

Hexadecanoic acid or palmitic acid was the second dominant compound of this essential oil with a value of 15.44%. In previous studies, this composition was found in the Gonbarf region of East Azerbaijan in the amount of 2.34% [36, 45], and in the amount of 1.14% in Noushabad, Kashan ([25]) and it was reported from Qamsar, Kashan as 2.30% [26]. The 16-carbon long-chain palmitic acid is the most common saturated fatty acid in the human body and can be obtained from the diet or synthesized endogenously from other fatty acids, carbohydrates, and amino acids [46, 47]. Palmitic acid is an essential component of cell membrane, secretory lipids and transport and plays an important role in protein palmitoylation and palmitoylated signal molecules [48]. Palmitic acid acts as an energy source and a signaling molecule involved in the regulation of various diseases [49]. It can reduce the malignant proliferation of hepatocellular carcinoma in mouse xenografts and thereby reduce the invasion of cancer cells [50], inhibits cell proliferation and invasion, and induces apoptosis of human gastric cancer cells [51].

The third dominant compound of this essential oil was octadecanoic acid or stearic acid (10.17%), which was recorded only from Isfahan in the amount of 4.09% for this essential oil [36]. Chemically, stearic acid is an 18-carbon chain fatty acid, which is the second most common naturally occurring saturated fatty acid after palmitic acid [52]. Stearic acid and its derivatives have been studied to prepare pharmaceutical, cosmetic and food formulations. Stearic acid has many health benefits when taken orally or applied topically to the skin [53].

Citronellol was the fourth dominant compound of this essential oil with a value of 7.37%. Based on previous studies, citronellol has been reported as the dominant and first compound of *R.* × *damascena* essential oil in different regions of Iran. For example, from Darab region of Fars province with 41.44% [34], from Kashan with 37.1% Hadipour et al. [32], from Kamoo with 36.70% [26], from Chaghakhor in Chaharmahal and Bakhtiari province with 41.78% [31], from Saadat Shahr in Fars province with 34.4% Yavari et al. [28], and from Majarshin with 45.2% [45] has been registered. The lowest amount of this compound in different areas of Kashan is reported from Sedeh village with 9.18% [26]. In other regions of Iran, the lowest amount of citronellol is from Shahrekord region with a value of 7. 1% was reported as a non-major compound of *R.* × *damascena* essential oil. Citronellol is an oxygenated monoterpene found in the essential oil of plants of the genus *Cymbopogon* and has several pharmacological activities. Among other things, it has the properties of lowering blood pressure, pain relief, vasorelaxant, anti-inflammatory, anticonvulsant, antidepressant, antibacterial, fungicidal, and antidiabetic [54–57].

Nonadecane alkane hydrocarbon with 3.70% was the fifth dominant compound of this essential oil. Nonadecane with 11.2% from Saadat Shahr [28], 16.44% from Darab [34], and 20.46% from Qazi Jahan [45] as the second dominant compound of *R.* × *damascene* essential oil has been reported. Hadipour et al. [32] recorded this compound as the third dominant compound (11.5%) of this essential oil. Davoodi et al. [31] from different regions reported different amounts of nonadecane for *R.* × *damascene* essential oil, which was in Chamchank region (34.17%) as the first compound and in Chaghakhor region (0.1%) as a minor compound.

Geraniol, which is considered one of the aroma indicators of *R.* × *damascene* essential oil, was observed in a small amount (1.58%) in the present study. Similarly, [45] reported the low amount of this compound from Kordabad (0.94%) and Gonbarf (2.30%) regions. This composition has been recorded by Hadipour et al. [32] with 12.7% and by Yavari et al. [28] with 26.5% as the second dominant composition of this essential oil. The best aroma of *R.* × *damascene* essential oil is when the geraniol/citronellol ratio is greater than 1.25 [58]. In the present study, the ratio of geraniol/citronellol is 4.66. Geraniol is an aliphatic monoterpene structure that has a functional alcohol group in its organic composition. The biological and medicinal properties of this compound include anti-diabetic, anti-tumor, anti-depressant, antimicrobial, antioxidant and anti-inflammatory activity [59]. Studies show that geraniol and citronellol have a calming effect and reduce anxiety, stress and depression [60].

### Antimicrobial activity of essential oil

The ANOVA results showed that there was a significant difference between the inhibition zone of *R. x damascena* essential oil and the control antibiotics against the different studied strains (P ≤ 0.01) (Table 2). The highest zone of inhibition of *R. x damascena* essential oil was against *Candida albicans* (ATCC 10231) (~ 9.5 mm), which was three times weaker compared to the antibiotic nystatin (~ 33 mm). Odyntsova et al. [61] reported the diameter of the inhibition zone of *R. x damascena* essential oil against *C. albicans* as 33.3 mm, which is not consistent with our results. In previous studies on this essential oil in different regions of Kashan, no inhibition zone for this yeast was observed [25, 26, 36]. The existence of differences in the diameter of the inhibition zone of the essential oil of a species can be due to the differences in the composition of the essential oil, which is probably caused by the growing places of the plant that produces the essential oil and as a result, different chemical profiles. It seems that the predominance of acidic compounds such as oleic acid, palmitic acid, stearic acid as well as the oxygenated monoterpene compound citronellol is one of the main factors in creating this inhibition zone in our study. The antimicrobial activity of essential oils is not only caused by the dominant compounds, and the synergistic effect with small compounds can also be one of the other reasons for this. Therefore, the small amount of geraniol and eugenol are also possible factors of this inhibitory activity. The effect of geraniol [59] and citronellol [62], eugenol [63] has been confirmed against *C. albicans*. Antifungal activities of fatty acids have been confirmed and their activity spectrum is variable based on degree of saturation, carbon chain length and double bond orientation [64].

**Table 2:** Antimicrobial activity of *R. x damascena* essential oil

Standard strains	Essential oil			Rifampin		Gentamicin		Nystatin	
	**IZ (mm)**	**MIC (μg/mL)**	**MBC (μg/mL)**	**IZ (mm)**	**MIC (μg/mL)**	**IZ (mm)**	**MIC (μg/mL)**	**IZ (mm)**	**MIC (μg/mL)**
*B. subtilis*	8.50 ± 0.50^c^	2000	4000	19.00 ± 0.00^b^	31.25	30.00 ± 0.00^a^	3.90	NA	NA
*S. aureus*	8.00 ± 0.00^c^	4000	4000	21.00 ± 0.00^a^	31.25	27.00 ± 0.00^b^	1.95	NA	NA
*S. epidermidis*	ND	4000	4000	27.00 ± 0.00^b^	1.95	45.00 ± 0.00^a^	1.95	NA	NA
*E. coli*	ND	8000	8000	11.00 ± 0.00b	3.90	20.00 ± 0.00a	3.90	NA	NA
*K. pneumoniae*	ND	4000	8000	8.00 ± 0.00^b^	15.63	17.00 ± 0.00^a^	3.90	NA	NA
*P. aeruginosa*	ND	2000	4000	ND	31.25	20.00 ± 0.00^a^	7.81	NA	NA
*S. paratyphi-A*	ND	2000	4000	8.00 ± 0.00^b^	15.63	18.00 ± 0.00^a^	3.90	NA	NA
*Sh. dysenteriae*	ND	8000	8000	9.00 ± 0.00^b^	15.63	17.00 ± 0.00^a^	3.90	NA	NA
*A. baumannii*	ND	500	1000	8.00 ± 0.00^c^	7.81	17.00 ± 0.00^a^	3.90	NA	NA
*P. mirabilis*	9.00 ± 0.00^b^	2000	4000	9.00 ± 0.00^b^	15.63	20.00 ± 0.00^a^	31.25	NA	NA
*C. albicans*	9.50 ± 1.50^b^	1000	1000	NA	NA	NA	NA	33.00 ± 0.00^a^	125

IZ: inhibition zone in diameter around the well impregnated with *R. x damascena* essential oil for each microorganism. Inhibition zone values include the well diameter (6.0 mm).ND: no inhibitory effect was detected. NA: no activity. Values with different letters are statistically different (Duncan, *p* ≤ 0.05)

As the most common invasive fungal pathogen, *C. albicans* can cause superficial and life-threatening systemic infections with high mortality [65]. For overcome these limitations, several studies have sought to identify new treatment strategies for candidiasis, and the present study can be a possible natural option in this regard.

The findings showed that the strongest inhibitory activity of *R. x damascena* essential oil against Gram-negative *Proteus mirabilis* (ATCC 43071) was with the diameter of the inhibition zone (~ 9 mm), which was equal to the strength of rifampin (~ 9 mm) and one time weaker than gentamicin (~ 20 mm). To the best of our knowledge, there has been no report on the activity of *R. x damascena* essential oil against *P. mirabilis*, and from this point of view, the present study is important, and it is the first report to introduce a possible potential against this bacterium. Echeverrigaray et al. [66] reported the anti-*P. mirabilis* effects of monoterpene compounds such as geraniol and cetronellol and found that the antimicrobial activity of a compound increases with the presence of an oxygen-containing functional group, which indicates the relationship between structure and biological activity. Devi et al. [67] found that eugenol showed excellent antibacterial activity against *P. mirabilis* by altering cell membrane integrity. Gram-negative bacteria have a more complex structure than Gram-positive bacteria, which includes a single layer of peptidoglycans surrounded by an outer layer containing proteins and lipopoly. This outer cell membrane is loaded with 20 saccharides and is hydrophilic in nature, thus limiting diffusion of the hydrophobic compound through LPS [68]. *P. mirabilis* causes urinary tract infections [69]. If this pathogenic bacterium reached the patient's blood, it would cause septicemia and bacteremia with relatively high mortality. In addition, it is developing resistance to numerous antibiotics, which poses a threat to public health worldwide [70]. Therefore, there is need to search for new alternatives to fight against such pathogenic bacteria.

Among the other inhibitory activities of this essential oil was the creation of the diameter of the inhibition zone against the Gram-positive *Bacillus subtilis* (ATCC 6633) (~ 8.5 mm), which is twice and three times compared to the antibiotics rifampin (~ 19 mm) and gentamicin (~ 30 mm), respectively. had acted weaker. Yi et al. [71] reported the inhibition zone diameter of *R.* × *damascena* essential oil against this bacterium as 44.73 mm, which is contrary to the present results. It seems that the predominant compounds of cetronol and fatty acids and small amounts of geraniol are the main factors of this antibacterial activity. Yi et al. [71] recorded the inhibition zone diameter of citonolol and geraniol against *B. subtilis* as 19.41 and 19.73 mm, respectively. Firoozabad and Nasr [72] reported the diameter of the inhibition zone of oleic acid against *B. subtilis* as 9 mm. *B. subtilis* is non-pathogenic but can contaminate food and is considered as an opportunistic pathogen among immunocompromised individuals [73].

Another inhibitory activity of this essential oil was against Gram-positive *Staphylococcus aureus* (ATCC 29737) with a diameter of about 8 mm, which was about three times weaker than compared to antibiotics rifampin (~ 21 mm) and gentamicin (~ 27 mm). Similarly, the diameter of the inhibition zone against this bacterium has been reported to be 11.3 mm [36, 61]. The largest diameter of the inhibition zone of *R.* × *damascena* essential oil against *S. aureus* (30.11 mm) by Yi et al. [71] has been recorded. The inhibitory activity of citronellol and geraniol with the diameter of the inhibition zone of 15 mm by Jirovetz et al. [74] and with the diameter of 19.56 and 18.75 mm by Yi et al. [71] against *S. aureus* has been confirmed. The zone of inhibition of oleic acid against *S. aureus* has been reported to be 7 mm. *S. aureus* readily adapts its metabolic and pathogenic responses in different tissues, causing both superficial (eg, folliculitis) and invasive (eg, osteomyelitis; Balasubramani) infections [75]. Widespread microbial resistance has highlighted the need to develop additional treatments against this bacterium [76].

Based on the results of the minimum inhibitory concentration and lethality, the value of MIC and MBC/MFC of *R. x damascena* essential oil against different strains ranged from 500 to 8000 μg/mL and from 1000 to 8000 μg/mL, respectively, which compared to positive controls performed very poorly. The lowest MIC value of this essential oil against Gram-negative *Acinetobacter baumannii* (ATCC BAA-747)was equal to 500 μg/mL, which had a very weak effect compared to control antibiotics. In previous studies, there is no report on the inhibitory or lethal activity of *R. x damascena* essential oil against this bacterium, and our study is the first report of this activity.

## Conclusion

The essential oil of *R.* × *damascena* is a high-value natural product with its unique qualitative properties, which was evaluated in this study for the first time from Azaran region, Kashan, Iran. This essential oil was white in color with a relatively high yield (~ 0.16 percent). Qualitatively, for the first time, more than 73.69% of *R.* × *damascena* essential oil consisted of saturated and unsaturated fatty acids (oleic acid, palmitic acid and stearic acid) and it has the strongest inhibitory activity against Gram-negative *P. mirabilis* with the strength of rifampin. The results show that *R.* × *damascena* essential oil may be used as a promising natural agent in the development of treatment for infectious diseases, especially urinary tract infection. Therefore, conducting more studies, especially in the clinical phase, may lead to the discovery of new drug leads with the potential treatment of various diseases.

## Data Availability

The datasets generated and/or analysed during the current study are available from the corresponding author on reasonable request.

## References

[1] Jowkar A, Kermani M, Kafi M, Mardi M, Hosini ZS, Koobaz P (2009). Cytogenetic and flow Cytometry analysis of Iranian *Rosa* spp. Floriculture Ornamental Biotech.

[2] Hosseini B, Shameh Sh, Alirezalu A (2018). Evaluation of distribution and phytochemical diversity of roses species (Rosa spp.) in Northwest of Iran. J Plant Product Res.

[3] Zgheib R, Najm W, Azzi-Achkouty S, Sadaka C, Ouaini N, Beyrouthy ME (2020). Essential oil chemical composition of Rosa corymbifera Borkh., Rosa phoenicia Boiss. and Rosa damascena Mill. from Lebanon. J Essential Oil Bearing Plants.

[4] Galal TM, Al-Yasi HM, Fawzy MA, Abdelkader TG, Hamza RZ, Eid EM (2022). Evaluation of the Phytochemical and Pharmacological Potential of Taif’s Rose (Rosa damascena Mill var. trigintipetala) for Possible Recycling of Pruning Wastes. Life.

[5] Deltalab B, Kaviani B, Kulus D (2023). In vitro propagation of oil-bearing Rosa damascena using phloroglucinol: A protocol for rapid and high-quality shoot multiplication and rooting. Ind Crops Prod.

[6] Andalib S, Vaseghi A, Vaseghi G, Naeini AM (2011). Sedative and hypnotic effects of Iranian traditional medicinal herbs used for treatment of insomnia. Excli J.

[7] Labban L, Thallaj N (2020). The medicinal and pharmacological properties of Damask Rose (*Rosa damascene* L.): A review. International Journal of Herbal Medicine.

[8] Nasiri M, Torkaman M, Feizi SH, Shamloo BB, M.  (2021). Effect of aromatherapy with Damask rose on alleviating adults’ acute pain severity: A systematic review and meta-analysis of randomized controlled trials. Complement Ther Med.

[9] Beheshti F, Ahmadabady S, Baghcheghi Y, Anaeigoudari A, Hosseini M (2021). A mini review of neuropharmacological effects of Rosa damascena Herrm. Pharmaceutical Sciences.

[10] Afsari Sardari F, Mosleh G, Azadi A, Mohagheghzadeh A, Badr P (2019). Traditional and recent evidences on five phytopharmaceuticals from *Rosa × damascene* Herrm. Res J Pharmacogn.

[11] Arvin P, Firuzeh R (2022). Ethnobotany of medicinal plants in Razo-Jargalan district in North Khorasan province. Iranian Journal of Medicinal and Aromatic Plants Research.

[12] Sabzi nojedeh M, Amani M, Younessi Hamzekhanlu M, badri&lrm L, Fathizadeh O, Sheidai Karkaj E (2021). Medicinal plants with therapeutic uses in indigenous communities located in the foothills of Sabalan (Case study: Meshginshahr city, Ardabil province). J Range and Watershed Manag.

[13] Ghavam M, Kiani S (2018). Ethnobutanical analysis of medicinal plants in Kashan. J Nat Eco Iran.

[14] Mohammadi F, Ahmadi A, Mokhtarpour M (2022). Quantitative Evaluation of Biodiversity of Medicinal Plants and Their Use in Darab City. Iranian Journal Of Antheropology.

[15] Amirahmadi A, Ghamari F (2023). Evaluation of the most commonly used medicinal plants purchased from the traditional markets of Semnan city, in order to identify plants that need protection. Ethnobiology and Conservation.

[16] Mehrabani M, Mahdavi Meymand Z, Mirtajadini M (2013). Collecting and identifying a selection of wild plants of Baft township (Iran, Kerman province) and study of their traditional uses. Jiitm.

[17] Khare S, Gupta M, Cheema HS, Maurya AK, Rout P, Darokar MP, Pal A (2018). Rosa damascena restrains Plasmodium falciparum progression in vitro and impedes malaria pathogenesis in murine model. Biomed Pharmacother.

[18] Hamedi B, Pirbalouti A.G., Rajabzadeh F (2022). Manures, vermicompost, and chemical fertilizer impacts on the yield and volatile compounds of the damask rose. Industrial Crops and Products.

[19] Mohsen E, Younis IY, Farag MA (2020). Metabolites profiling of Egyptian Rosa damascena Mill. flowers as analyzed via ultra-highperformance liquid chromatography-mass spectrometry and solid-phase microextraction gas chromatography-mass spectrometry in relation to its anti-collagenase skin effect. Ind Crops Prod..

[20] Akram M, Riaz M, Munir N, Akhter N, Zafar S, Jabeen F, Ali Shariati M, Akhtar N, Riaz Z, Altaf SH, Daniyal M, Zahid R, Said Khan F (2020). Chemical constituents, experimental and clinical pharmacology of Rosa damascena: a literature review. J Pharm Pharmacol.

[21] Tambe E, Gotmare SR (2016). Study of Variation and Identification of Chemical Composition in Rosa Species Oil Collected from Different Countries. IOSR Journal of Applied Chemistry.

[22] Panahi M (2023). A review on phylogeny of Damask Rose (Rosa × damascena). Eco-phytochemical Journal of Medicinal Plants.

[23] Adams RP (2011). Identification of Essential Oils by Ion Trap Mass Spectroscopy.

[24] CLSI. Clinical and Laboratory Standard Institute. Performance standards for antimicrobial disk susceptibility testing: Approved standard: National Committee for Clinical Laboratory Standards. 2012;29:1-76.

[25] Ghavam M (2021). Relationships of irrigation water and soil physical and chemical characteristics with yield, chemical composition and antimicrobial activity of Damask rose essential oil. PLoS ONE.

[26] Ghavam M, Afzali A, Manconi M, Bacchetta G, Manca ML (2021). Variability in chemical composition and antimicrobial activity of essential oil of Rosa × damascena Herrm. from mountainous regions of Iran. Chem Biol Tech Agri.

[27] Yousefi B, Jaimand K (2018). Chemical variation in the essential oil of Iranian Rosa damascena landraces under semi-arid and cool conditions. International Journal of Horticultural Science and Technology.

[28] Yavari A, Raheb A, Norouzi M (2023). Comparison in essential oil of Rosa damascena Mill. during harvest period under calcareous soil conditions. J Plant Process Function.

[29] Mohammadnezhad Ganji SM, Moradi H, Ghanbarzadeh A, Akbarzadeh M (2014). Investigating the altitude effect on the quantity and quality of the essential oil in *Rosmarinus officinalis* in Mazandaran Province. Eco-Phytochemical Journal of Medical Plants.

[30] Arianfar M, Akbarinodehi D, Hemati K, Rostampoor M (2018). Effects of altitude and aspect on efficiency of producing essence and phytochemical properties of Artemisia aucheri Boiss and Artemisia sieberi Besser in South Khorasan rangelands..

[31] Davoodi K, Ghasemi Pirbalouti A, Malekpoor F (2019). Phytochemical diversity of Rosa damascene Mill populations in the north of Chaharmahal and Bakhtiari province. Journal of Medicinal Herbs, "J. Med Herb" (Formerly known as Journal of Herbal Drugs or J. Herb Drug).

[32] Hadipour E, Kafash MR, Emami SA, Asili J, Boghrati Z, Tayarani-Najaran Z (2023). Evaluation of anti-oxidant and antimelanogenic effects of the essential oil and extracts of Rosa× damascena in B16F10 murine melanoma cell line. Iran J Basic Med Sci.

[33] Shahbazi K, Shahbazi K, Yousefi B, Safari H (2022). Evaluation of Chemical Compounds of Essential Oil in Damask Rose (Rosa damascena Mill.) Accessions. J Med Plants By-product.

[34] Mosleh  G, Badr P, Azadi A, Sardari FA, Iraji A, Khademian S (2021). Quality control of Rosa × damascena flowers from Layzangan of Fars province in Iran. J Med Plants.

[35] Melito S, Petretto GL, Podani J, Foddai M, Maldini M, Chessa M, Pintore G (2016). Altitude and climate influence *Helichrysum italicum* subsp. microphyllum essential oils composition. Ind Crops Prod.

[36] Ghavam M, Afzali A, Manca ML (2021). Chemotype of damask rose with oleic acid (9 octadecenoic acid) and its antimicrobial effectiveness. Sci Rep.

[37] Schwingshackl L, Hoffmann G (2012). Monounsaturated Fatty Acids and Risk of Cardiovascular Disease: Synopsis of the Evidence Available from Systematic Reviews and Meta-Analyses. Nutrients..

[38] Martinez M, Mougan I (1998). Fatty Acid Composition of Human Brain Phospholipids During Normal Development. J Neurochem.

[39] Hamazaki K, Hamazaki T, Inadera H (2012). Fatty Acid Composition in the Postmortem Amygdala of Patients with Schizophrenia, Bipolar Disorder, and Major Depressive Disorder. J Psychiatr Res.

[40] Dilika F, Bremner PD, Meyer JJM (2000). Antibacterial activity of linoleic and oleic acids isolated from Helichrysum pedunculatum: a plant used during circumcision rites. Fitoterapia.

[41] Chen J, Li Q, Zhang Y, Yang P, Zong Y, Qu S (2011). Oleic Acid Decreases the Expression of a Cholesterol Transport-Related Protein (NPC1L1) by the Induction of Endoplasmic Reticulum Stress in CaCo-2 Cells. J. Physiol. Biochem..

[42] Bhattacharjee B, Pal PK, Chattopadhyay A, Bandyopadhyay D (2020). Oleic Acid Protects against Cadmium Induced Cardiac and Hepatic Tissue Injury in Male Wistar Rats: A Mechanistic Study. Life Sci..

[43] Linos A, Kaklamanis E, Kontomerkos A, Koumantaki Y, Gazi S, Vaiopoulos G, Tsokos GC, Kaklamanis P (1991). The effect of olive oil and fish consumption on rheumatoid arthritis–a case control study. Scand J Rheumatol.

[44] Solanas M, Hurtado A, Costa I, Moral R, Menendez JA, Colomer R, Escrich E (2002). Effects of a high olive oil diet on the clinical behavior and histopathological features of rat DMBAinduced mammary tumors compared with a high corn oil diet. Int J Oncol.

[45] Seyed Hajizadeh H, Ebadi B, Morshedloo MR, Abdi Ghazijahani A (2021). Morphological and phytochemical diversity among some Iranian Rosa damascena Mill. landraces. J Ornamental Plants.

[46] Carta G, Murru E, Banni S, Manca C (2017). Palmitic acid: physiological role, metabolism and nutritional implications. Front Physiol.

[47] Pascual G, Domínguez D, Elosúa-Bayes M, Beckedorff F, Laudanna C, Bigas C, Douillet D, Greco C, Symeonidi A, Hernández I (2021). Dietary palmitic acid promotes a prometastatic memory via Schwann cells. Nature.

[48] Agostoni C, Moreno L, Shamir R (2016). Palmitic acid and health: Introduction. Crit Rev Food Sci Nutr.

[49] Liu XZ, Rulina A, Choi MH, Pedersen L, Lepland J, Takle ST (2022). C/EBPB-dependent adaptation to palmitic acid promotes tumor formation in hormone receptor negative breast cancer. Nat. Commun..

[50] Lin L, Ding Y, Wang Y, Wang Z, Yin X, Yan G (2017). Functional lipidomics: Palmitic acid impairs hepatocellular carcinoma development by modulating membrane fluidity and glucose metabolism. Hepatology..

[51] Yu X, Peng W, Wang Y, Xu W, Chen W, Huang L, Xu Y (2023). Palmitic Acid Inhibits the Growth and Metastasis of Gastric Cancer by Blocking the STAT3 Signaling Pathway. Cancers.

[52] Ye S, Noda H, Morita S, Uosaki K, Osawa M (2003). Surface Molecular structures of langmuir− blodgett films of stearic acid on solid substrates studied by sum frequency generation spectroscopy. Langmuir.

[53] Hu FQ, Zhao MD, Yuan H, You J, Du YZ, Zeng S (2006). A novel chitosan oligosaccharide–stearic acid micelles for gene delivery: Properties and in vitro transfection studies. Int J Pharm.

[54] Santos MR, Moreira FV, Fraga BP, Souza DPD, Bonjardim LR, Quintans-Junior LJ (2011). Cardiovascular effects of monoterpenes: a review. Rev Bras.

[55] Bastos JF, Moreira ÍJ, Ribeiro TP, Medeiros IA, Antoniolli AR, De Sousa DP, Santos MR (2010). Hypotensive and vasorelaxant effects of citronellol, a monoterpene alcohol, in rats. Basic Clin Pharmacol Toxicol.

[56] Brito RG, Guimarães AG, Quintans JS, Santos MR, De Sousa DP, Badaue-Passos D, Quintans LJ (2012). Citronellol, a monoterpene alcohol, reduces nociceptive and inflammatory activities in rodents. Journal of natural medicines.

[57] Qneibi M, Jaradat N, Emwas N (2019). Effect of geraniol and citronellol essential oils on the biophysical gating properties of AMPA receptors. Applied Sciences.

[58] Başer KHC (1992). Turkish Rose Oil. Perfum. Flavor.

[59] Lira MHPD, Andrade Júnior FPD, Moraes GFQ, Macena GDS, Pereira FDO, Lima IO (2020). Antimicrobial activity of geraniol: An integrative review. J Essent Oil Res.

[60] Heghes SC, Vostinaru O, Rus LM, Mogosan C, Iuga CA, Filip L (2019). Antispasmodic e_ect of essential oils and their constituents: A review. Molecules.

[61] Odyntsova V, Denysenko O, Shkopynska T, Mozul V, Polishchuk N, Aksonova I, et al. Chemical composition and antimicrobial activity of Rosa Damascena mill. (variety rainbow) from clonal micropropagation. ScienceRise: Pharmaceutical Science. 2023;89–96.

[62] Zore G, Thakre A, Jadhav S, Karuppayil S (2011). Terpenoids inhibit Candida albicans growth by affecting membrane integrity and arrest of cell cycle. Phytomedicine.

[63] Schmidt E, Jirovetz L, Wlcek K, Buchbauer G, Gochev V, Girova T, Stoyanova A, Geissler M (2007). Antifungal Activity of Eugenol and Various Eugenol-Containing Essential Oils against 38 Clinical Isolates of *Candida albicans*. Journal of Essential Oil-Bearing Plants.

[64] Mattanna P, Da Rosa PD, Poli J, Richards NSPS, Daboit TC, Scroferneker ML, Valente P (2014). Lipid profile and antimicrobial activity of microbial oils from 16 oleaginous yeasts isolated from artisanal cheese. Rev Bras Bioci.

[65] Wang Y, Yan H, Li J, Zhang Y, Wang Z, Sun S (2023). Antifungal activity and potential mechanism of action of caspofungin in combination with ribavirin against Candida albicans. Int J Antimicrob Agents.

[66] Echeverrigaray S, Michelim L, Delamare APL, Andrade CP, da Costa SOP, Zacaria J (2008). The effect of monoterpenes on swarming differentiation and haemolysin activity in Proteus mirabilis. Molecules.

[67] Devi KP, Sakthivel R, Nisha SA (2013). Eugenol alters the integrity of cell membrane and acts against the nosocomial pathogen *Proteus mirabilis*. Arch Pharm Res.

[68] Baygan A, Safaeian S, Shahinfar R, Khoshkhoo Z (2022). Evaluation of antibacterial effect of Ziziphora clinopodioides essential oil in North Khorasan region on gram-negative bacteria Salmonella typhimurium in vitro. FSCT.

[69] Scavone P, Iribarnegaray V, González MJ, Navarro N, Caneles-Huerta N, Jara-Wilde J, Härtel S, Zunino P (2023). Role of Proteus mirabilis flagella in biofilm formation. Rev Argent Microbiol..

[70] Elekhnawy E, Almurshedi AS, Abdelkader DH, El-Masry TA, Aldosari BN, El-Bouseary MM (2023). Green synthesised zinc oxide nanoparticles reveal potent in vivo and in vitro antibacterial efficacy against Proteus mirabilis isolates. Int J Pharm..

[71] Yi F, Sun J, Bao X, Ma B, Sun M. Influence of molecular distillation on antioxidant and antimicrobial activities of rose essential oils. Lwt. 2019;102:310–6.

[72] Firoozabad MSM, Nasr MM (2022). Antimicrobial activities of microbial essential fatty acid against foodborne pathogenic bacteria. Iranian Journal of Microbiology.

[73] Ghavam, M., Markabi, F.S. Evaluation of Yield, Chemical Profile, and Antimicrobial Activity of *Teucrium polium* L. Essential Oil Used in Iranian Folk Medicine. *Appl Biochem Biotechnol* (2024). 10.1007/s12010-023-04847-6 10.1007/s12010-023-04847-6 38194183

[74] Jirovetz L, Eller G, Buchbauer G, Schmidt E, Denkova Zapryana, Stoyanova A (2006). Chemical composition, antimicrobial activities and odor descriptions of some essential oils with characteristic floral-rosy scent and of their principal aroma compounds. Rec. Res. Dev. Agron. Horticult..

[75] Potter AD, Butrico CE, Ford CA, Curry JM, Trenary IA, Tummarakota SS (2020). Host nutrient milieu drives an essential role for aspartate biosynthesis during invasive Staphylococcus aureus infection. Proc Natl Acad Sci U S A.

[76] Ventola CL (2015). The antibiotic resistance crisis: causes and threats. P T.

